# A Selective Chromogenic Medium for Detecting Meropenem-Resistant *Pseudomonas aeruginosa* in Respiratory Samples

**DOI:** 10.3390/antibiotics14050480

**Published:** 2025-05-09

**Authors:** Carmen Cintora Mairal, Guillermo Martín-Gutiérrez, Ángel Rodríguez-Villodres, José Miguel Cisneros, José Antonio Lepe, José Manuel Ortiz de la Rosa

**Affiliations:** 1 Clinical Unit of Infectious Diseases, Microbiology and Parasitology, University Hospital Virgen del Rocío, 41013 Seville, Spain; carmencintora20@gmail.com (C.C.M.); anrovi1797@gmail.com (Á.R.-V.); jmcisnerosh@gmail.com (J.M.C.); josealepe@gmail.com (J.A.L.); 2 Institute of Biomedicine of Seville (IBiS), University Hospital Virgen del Rocío, Higher Council for Scientific Research (CSIC), University of Seville, 41013 Seville, Spain; 3 Centro de Investigación Biomédica en Red de Enfermedades Infecciosas (CIBERINFEC), 28029 Madrid, Spain; 4 Department of Health Sciences, Loyola Andalucía University, 41704 Sevilla, Spain; 5 Department of Medicine, Faculty of Medicine, University of Seville, 41004 Seville, Spain; 6 Department of Microbiology, Faculty of Medicine, University of Seville, 41009 Seville, Spain

**Keywords:** meropenem, empiric treatment, *Pseudomonas aeruginosa*

## Abstract

**Background/Objectives**: Meropenem is widely used to treat Pseudomonas aeruginosa infections; however, the pathogen’s increasing resistance compromises its efficacy. In this study, we aimed to develop a selective culture medium for detecting the presence of meropenem-resistant *Pseudomonas aeruginosa* in respiratory specimens within 24 h. **Methods**: The medium’s performance was challenged using a collection of 130 clinical *Pseudomonas aeruginosa* strains (of which 85 were meropenem-susceptible, 14 were meropenem-intermediate, and 21 were meropenem-resistant). Subsequently, clinical validation was carried out using 130 respiratory samples. **Results**: The selective medium demonstrated excellent sensitivity (average 98.7%) and specificity (average 90%) across bacterial concentrations ranging from 1 × 10^4^ to 1 × 10^8^ CFU/mL, and a high negative predictive value (average 99.2%) compared to the broth microdilution (BMD) method. Clinical validation with bronchoalveolar lavage (BAL) and tracheobronchial aspirate (TBA) clinical specimens (N = 130) revealed a strong performance, with 92,3% categorical agreement. **Conclusions**: This method accelerates susceptibility testing, is user-friendly, and delivers reliable results, contributing to the optimization of empirical treatment for respiratory tract infections.

## 1. Introduction

Meropenem (MER) is frequently used as a first-line treatment for infections caused by *Pseudomonas aeruginosa* due to its broad-spectrum activity and efficacy against Gram-negative bacteria. The Infectious Diseases Society of America (IDSA) and the American Thoracic Society (ATS) recommend piperacillin–tazobactam (TZP), cefepime (FEP)/ceftazidime (CAZ), or MER for the empiric treatment of ventilator-associated pneumonia and hospital-acquired pneumonia in units where double antipseudomonal coverage is appropriate [1].

However, the rapid emergence of resistance in *P. aeruginosa* is cause for significant concern, particularly in intensive care units (ICUs). Prolonged hospital stays, invasive procedures, mechanical ventilation, and extensive antibiotic exposure are key factors that contribute to the selection and dissemination of resistant strains in ICU patients. These factors increase the risk of colonization and infection with multidrug-resistant organisms, including *P. aeruginosa*. The frequent use of broad-spectrum antibiotics, such as meropenem, in these settings further exacerbates the problem by applying selective pressure that promotes the survival and proliferation of resistant strains [2,3,4].

The mechanisms of meropenem resistance in *P. aeruginosa* are diverse and include overexpression of efflux pumps (e.g., MexAB-OprM), loss of outer membrane porins (e.g., OprD downregulation), and production of carbapenemases (e.g., VIM, IMP, KPC, and NDM). These resistance mechanisms significantly limit treatment options, often requiring combination therapies with agents such as amikacin or colistin to improve clinical outcomes. The increasing prevalence of carbapenem-resistant *P. aeruginosa* underscores the need for rapid diagnostic tests to detect resistance mechanisms promptly and inform appropriate therapeutic guidelines [5,6]. In recent years, multiplex PCR-based molecular techniques have been developed to identify respiratory pathogens directly from the samples [7]. However, molecular techniques are limited by discrepancies between genotype and phenotype, especially in *P. aeruginosa*, which could lead to misinterpretation of the results [8].

Studies have associated inappropriate initial antimicrobial therapy for *P. aeruginosa* infections with increased mortality and longer hospital stays, emphasizing the need for rapid resistance testing to ensure timely and effective treatment adjustments. Rapid identification of resistance could help optimize antimicrobial therapy, reduce the use of ineffective antibiotics, and improve patient outcomes, and could facilitate antimicrobial stewardship by minimizing unnecessary use of broad-spectrum antibiotics, thereby reducing the selection pressure on resistant strains [9,10,11,12]. Several studies emphasize rapid diagnostic tests’ critical role in improving outcomes for patients with *P. aeruginosa* pneumonia. Riccobene et al. (2024) highlight that inadequate empirical therapy and delays in initiating newer antibiotics were associated with significantly worse hospital outcomes, including higher in-hospital mortality and longer hospital stays in patients with *P. aeruginosa* infections underscoring the importance of quickly detecting resistance mechanisms to guide treatment and improve clinical outcomes [13]. Similarly, Radulescu and Gant (2023) describe how rapid molecular diagnostic platforms, such as multiplex PCR, can replace empirical antibiotic choices with evidence-based targeted therapies within clinically actionable timeframes, thereby improving patient outcomes and antimicrobial stewardship [14]. Lynch and Zhanel (2022) also reported an association between inadequate initial therapy and higher mortality, particularly in ventilator-associated pneumonia, and advocated for rapid resistance testing to optimize therapy [15]. Finally, in the IDSA and ATS guidelines, Kalil et al. (2016) also identified an association between inappropriate therapy and increased mortality and recommend rapid diagnostic tests to ensure timely adjustments to treatment [1].

**Figure 1:**
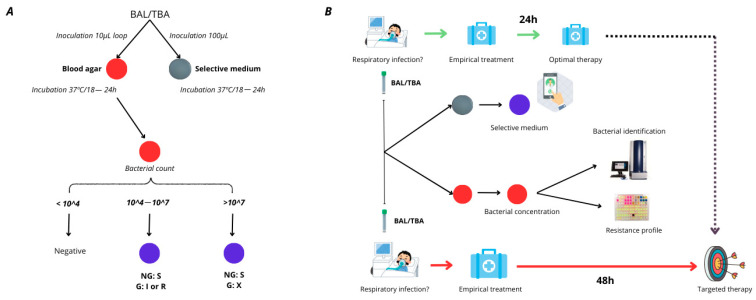
(**A**) Diagnostic algorithm designed for using the plates in a clinical setting. (**B**) Flowchart of the medium’s methodology and its possible application in clinical practice. BAL—bronchoalveolar lavage; TBA—tracheobronchial aspirate; G—growth; NG—no growth; S—susceptible; I—susceptible at increased exposure; R—resistant.

These studies collectively highlight the importance of timely and accurate resistance testing in improving the outcomes of patients with *P. aeruginosa* infections, particularly those involving the respiratory tract. Unfortunately, traditional antimicrobial susceptibility testing (AST) methods, such as broth microdilution or automated systems, can take 48–72 h, leading to treatment delays. Previously, our team developed a selective culture medium for screening TZP/FEP-resistant *P. aeruginosa* in respiratory samples [16]. In response to the diagnostic challenges posed by those samples, we identified the need to develop a selective medium for detecting meropenem-resistant *P. aeruginosa*. Thus, we aimed to design this medium based on the previously developed formulation, ensuring that the three empiric treatment options recommended by clinical guidelines for respiratory tract infections were addressed [16] [Figure 1].

## 2. Results

According to the Minimal Inhibitory Concentration (MIC) assay, this set included 85 MER-susceptible and 45 MER-resistant/intermediate (susceptible at increased exposure) isolates (Supplementary Table S1). Forty-four out of forty-five resistant isolates (97.8%) were detected on the meropenem medium. On the other hand, for susceptible strains, concordance decreased slightly due to the unexpected growth of some of the strains in the selective medium. The highest number of false positives was observed at a 1 × 10^8^ CFU/mL dilution, with 16 susceptible strains exhibiting growth on the medium. This was followed by the 1 × 10^7^ CFU/mL, 1 × 10^6^ CFU/mL, 1 × 10^5^ CFU/mL, and 1 × 10^4^ CFU/mL dilutions, which presented nine, seven, six, and six false positives, respectively.

These findings allowed us to establish the sensitivity and specificity of the medium for each of the tested concentrations. The sensitivity values exceeded 97% for all bacterial concentrations, with the majority reaching 100%. In contrast, specificity values varied across the different dilutions, with slight reductions; however, they remained at around 90% except at 1 × 10^8^ CFU/mL, where the value decreased to 81.2% (Table 1).

In the analysis of the 130 clinical strains collected from BAL and TBA, 13 *P. aeruginosa* isolates were recovered using the conventional methods employed by the Microbiology Service at the University Hospital Virgen del Rocío (Seville, Spain). All isolates obtained during the clinical sample validation were subsequently tested using the MicroScan WalkAway system (Beckman Coulter, Brea, CA, USA) and the gold standard method (BMD). When compared to the BMD reference method, the plates demonstrated a high degree of categorical agreement (CA), with a value of 92,3%. The reduction in CA was due to one major error (ME), which was a false-positive result (Supplementary Table S2). Despite the relatively small number of *P. aeruginosa* isolates recovered, the data obtained from the plates and BMD enabled the calculation of the sensitivity and specificity of the medium. However, the statistical analysis may not be sufficiently significant due to the limited number of *P. aeruginosa* isolates included during the validation process. The sensitivity was 100% (CI: (100%–100%) and the specificity was 80% (CI: 45–100%) (Table 2).

Moreover, the medium detected the growth of other meropenem-resistant Gram-negative bacteria, which were subsequently identified by MALDI-TOF (Bruker, Billerica, MA, USA) (Supplementary Table S2). After 24 h of incubation, no growth of competing microorganisms, including Gram-positive bacteria or fungi, was observed, indicating that the medium demonstrated strong specificity and selectivity for MER-resistant Gram-negative bacteria.

## 3. Discussion

In our work, we have developed a selective medium with excellent sensitivity and specificity. A study conducted in European ICUs, including Spain, found that 24.9% of *P. aeruginosa* isolates were resistant to meropenem, particularly in critically ill patients [17]. In 2019, another study reported that 17.3% of *P. aeruginosa* isolates were extensively drug-resistant (XDR), which included resistance to meropenem [18]. At our institution, *P. aeruginosa* was observed to be resistant to meropenem in 15% of cases in 2023, indicating that 15% of patients were at risk of receiving ineffective treatment. This issue could be mitigated using rapid antimicrobial resistance tests such as our selective culture medium. Moreover, the remaining 85% of patients would also benefit from early determination of antimicrobial susceptibility, allowing for timely de-escalation of therapy within the first 24 h. This strategy not only enhances antimicrobial stewardship but also minimizes unnecessary exposure to broad-spectrum antibiotics, thereby reducing the risk of selecting for resistant strains and improving overall clinical outcomes.

In 2021, Fournier et al. developed screening plates for carbapenem-resistant *Pseudomonas*; however, the cut-off was set to 10^1^–10^2^ CFU/mL and the breakpoint was 8 mg/L. As such, these places were unsuitable for respiratory samples, as a microorganism is considered an infection-causative agent at concentrations of >10^4^, and treatment differs when the MIC is between 2 and 8 mg/L [19]. Unlike the latter screening medium, our plates have been clinically validated for respiratory samples such as BAL and TBA, and were deemed particularly valuable for identifying resistant *P. aeruginosa* in intensive care unit patients, who are highly susceptible to such infections whilst undergoing mechanical ventilation. Furthermore, the common respiratory flora present in these samples did not interfere with the test, as it was effectively removed from the culture medium, ensuring complete selectivity. The implementation of this culture medium would be highly cost-effective, costing less than EUR 1 per plate. Given its ability to rapidly identify meropenem-resistant *P. aeruginosa*, its use could lead to significant cost savings by reducing the duration of ineffective treatments, preventing complications associated with inadequate therapy, and decreasing the length of hospital stays. Furthermore, by enabling early de-escalation of broad-spectrum antibiotics in susceptible cases, it facilitates more efficient resource utilization and helps mitigate the economic burden of antimicrobial resistance in healthcare settings.

However, certain limitations have been identified in terms of classifying strains as susceptible or resistant, particularly for strains that were susceptible at the MIC but showed growth in our medium. This primarily occurred at 1 × 10^8^ CFU/mL dilution, when the strains had an MIC of 2 (breakpoint). This observation is illustrated in Table 1, where at the aforementioned dilution, sensitivity reaches 100%, while specificity decreases to approximately 80%, indicating that a higher number of strains are classified as resistant compared to the reference method. The reduction in specificity at high bacterial concentrations could increase the number of falsely identified resistant strains, potentially resulting in overtreatment, which could represent a problem regarding antibiotic resistance and stewardship. Moreover, our selective medium cannot discriminate between resistant and intermediate strains (those that are susceptible when exposure is increased). Therefore, when our plates yield a positive result, the physician must decide whether to seek an alternative empirical treatment until the full resistance phenotype can be determined or, if no other options are available, administer a high dose of meropenem. Nevertheless, a positive aspect of this method is its high negative predictive value, which was almost 100% across all dilutions, indicating that it is highly unlikely for a resistant strain to fail to grow in our medium. This is crucial, as such an omission would represent a significant error, potentially leading to ineffective treatment or suboptimal doses for infected patients.

In conclusion, this method offers several notable advantages when combined with the usual culture media. Firstly, it significantly reduces the time required to determine the susceptibility to this first-line antibiotic in severe infections. Secondly, it is easy to prepare, making it a practical choice for clinical settings. Most importantly, it demonstrates high sensitivity and specificity, particularly with regard to its excellent negative predictive value, ensuring reliable identification of resistant strains. All of this may help to optimize the empirical treatment, where delaying the appropriate therapy is crucial for the outcome of the patients. Nevertheless, broader validation is necessary before this method can be implemented in clinical practice due to the limited number of *P. aeruginosa* isolates recovered in the validation step. Moreover, further studies are needed to assess its real impact on antimicrobial treatment strategies and patient outcomes in clinical practice.

## 4. Materials and Methods

Bacterial strains and study design: A total of 130 *P. aeruginosa* isolates were obtained from clinical samples, including bronchoalveolar lavage (BAL), tracheobronchial aspirates (TBA), and blood cultures. The samples were collected from the Microbiology Service at the University Hospital Virgen del Rocío in Seville, Spain. Of these, 21 isolates exhibited resistance to meropenem (MER), 14 exhibited intermediate resistance (susceptible at increased exposure), while 85 were classified as susceptible (Supplementary Table S1). To evaluate the plates under clinical conditions, additional BAL and TBA samples (130 specimens) were tested over a one-month period. The *P. aeruginosa* PAO1 strain served as a negative control, whereas one MER-resistant clinical isolate was used as a positive control. Other bacterial species, including *Stenotrophomonas maltophilia*, *Acinetobacter baumannii*, *Klebsiella pneumoniae*, and *Escherichia coli*, were incorporated to assess the chromogenic response of different species when cultured on plates.

Antimicrobial susceptibility testing: The minimum inhibitory concentration (MIC) of meropenem was determined using two methods: a MicroScan WalkAway system (Beckman Coulter, Brea, CA, USA) and the broth microdilution method (performed according to the European Committee on Antimicrobial Susceptibility Testing (EUCAST) guidelines) [20]. All susceptibility tests were conducted in triplicate, using freshly prepared plates and bacterial inocula for each experiment. MIC results were interpreted based on EUCAST clinical breakpoints, with isolates with MIC values of ≤2 mg/L classified as susceptible and those with MICs > 2 mg/L as resistant (although >2 to 8 mg/L is classified as intermediate, i.e., susceptible at increased exposure, we classify it as resistant).

Selective medium for MER resistance: For optimal bacterial screening, CLED agar (Biomerieux, Paris, France) was prepared according to the manufacturer’s instructions. The medium was supplemented with meropenem (Sigma-Aldrich, St. Louis, MO, USA) at a final concentration of 0.425 mg/L to facilitate the selective growth of resistant isolates. To minimize contamination by Gram-positive bacteria and fungi, vancomycin (Duchefa Biochemie, Haarlem, The Netherlands) and amphotericin B (Acros Organics, Fairlawn, NJ, USA) were incorporated at final concentrations of 20 μg/mL and 5 μg/mL, respectively. These additions effectively inhibited the growth of *Enterococcus* spp., *Streptococcus* spp., *Staphylococcus* spp., and fungal species. For visual differentiation of BIChromET medium [16], resazurin (1%) was included in the formulation. This allowed distinctions to be made between lactose-fermenting bacteria, which appeared as light beige colonies, and non-fermenting bacteria, which formed purple colonies. The stock solutions of meropenem, vancomycin, and amphotericin B were prepared as described in Table 1. CLED powder was dissolved in distilled water and sterilized by autoclaving at 121 °C for 30 min. Once the medium cooled to 56 °C, the antibiotic stock solutions were added (Table 3). The prepared plates were stored at 4 °C, protected from direct light, and remained viable for up to two weeks before use.

Evaluation assay: The sensitivity and specificity cut-off values for detecting MER-resistant *P. aeruginosa* were established at 1 × 10^3^ CFU/mL, considering the positive results of only the sample, of which the isolates were recovered onto the selective medium, plated at concentrations corresponding to >1 × 10^3^ CFU/mL. This cut-off value was fixed considering that BAL and TBA were positive when the 1 × 10^4^ CFU/mL bacteria were recovered from the clinical sample. Starting with a 0.5 McFarland standard (an inoculum of 1.5 × 10^8^ CFU/mL), serial 10-fold dilutions were made in 0.85% saline solution, and 100 μL aliquots of each dilution from 10^4^ to 10^8^ CFU/mL were plated onto the selective medium. To quantify the viable bacteria in each dilution step, tryptic soy agar plates were inoculated concomitantly with 100 μL of each suspension and incubated overnight at 37 °C. Viable colonies were counted the following day. When no growth was observed after 18 h, incubation was extended up to 48 h to assess the negativity of the culture. The medium was designed to detect resistant *P. aeruginosa* from 10^4^ to 10^8^ CFU/mL based on the IDSA guidelines [21]. It established that a BAL culture was positive when more than 10^4^ CFU/mL bacteria were recovered from said culture. After evaluation with a collection of 130 *P. aeruginosa*, the results were analyzed for each dilution of bacterial concentration used in this study.

Validation with clinical samples: To assess the performance of the selective medium in clinical settings, we analyzed 130 respiratory samples, including 62 BAL and 68 TBA specimens collected from the University Hospital Virgen del Rocío. Each sample (100 μL) was inoculated onto plates and incubated at 37 °C overnight. Following incubation, colonies displaying a distinct morphology, size, and pigmentation were selected for further characterization. Identification was performed via mass spectrometry, while antimicrobial resistance profiling for non-*P. aeruginosa* isolates was assessed using gradient strips (Supplementary Table S2). For *P. aeruginosa* isolates, meropenem resistance was confirmed through broth microdilution (BMD) testing. The results were interpreted according to the 2024 EUCAST breakpoints and compared with susceptibility data obtained from the Microbiology Service using the MicroScan WalkAway system (Beckman Coulter, Brea, CA, USA).

Statistical analysis: To evaluate the performance of the selective medium, specificity (the proportion of MER-susceptible isolates correctly identified) and sensitivity (the proportion of MER-resistant isolates correctly detected) were calculated for each bacterial concentration used in the evaluation. This analysis helped determine the medium’s limitations, assess its performance across different bacterial loads, and identify potential strategies to optimize its accuracy. Additionally, 95% confidence intervals (CIs), positive predictive value (PPV), and negative predictive value (NPV) were estimated to further assess the reliability of the medium. For the validation phase using clinical specimens (BAL and TBA), sensitivity, specificity, and their respective 95% CIs were calculated to determine the medium’s effectiveness in detecting meropenem resistance in *P. aeruginosa*. Broth microdilution was used as the gold standard method for comparison. During the clinical evaluation, true-positive results were considered when MER-resistant or intermediate *P. aeruginosa* (BMD MIC) growth was identified on the plates, while the absence of growth on the plates when MER-susceptible *P. aeruginosa* (BMD MIC) were present in the BAL/TBA samples led to a true-negative classification. Moreover, the endpoints were considered in categorical agreement when the results were in the same susceptibility category (regardless of the MIC) for *P. aeruginosa.* VME is classed as a very major error (false susceptibility), whereas ME is a major error (false resistance).

## Figures and Tables

**Table 1:** Performance of the selective medium.

		Bacterial Concentrations (CFU/mL)				
	Gold Standard	1 × 10^4^	1 × 10^5^	1 × 10^6^	1 × 10^7^	1 × 10^8^
Susceptible (N)	85	80	80	79	76	69
Resistance (N)	45	50	50	51	54	61
Sensitivity	-	97.8%	97.8%	97.8%	100%	100%
CI	-	93.5–100%	93.5–100%	93.5–100%	100–100%	100–100%
Specificity	-	92.9%	92.9%	91.8%	89.4%	81.2%
CI	-	87.5–98.3%	87.5–98.3%	86–97.6%	82.9–95.9%	72.9–89.5%
PPV	-	88%	88%	86.3%	83.3%	73.8%
NPV	-	98.7%	98.7%	98.7%	100%	100%

CI—95% confidence interval; PPV—positive predictive value; NPV—negative predictive value.

**Table 2:** Validation of the selective medium with respiratory samples.

	Selective Medium	BMD
Susceptible (N)	4	5
Resistance (N)	9	8
CA	92.3%	
Errors	MEs (N = 1)	
Sensitivity ^a^	100%	
CI	(100–100%)	
Specificity ^a^	80%	
CI	(45–100%)	

^a^ Sensitivity and specificity were exclusively calculated using the gold standard method (BMD) as a reference. CI—95% confidence interval; CA—categorical agreement; BMD—broth microdilution.

**Table 3:** Preparation of the medium.

Compound	Stock Solution	Quantity or Vol Added ^a^	Final Concentration
CLED agar medium		14.46 g	
Distilled water		400 mL	
Resazurin	1%	100 µL	0.00025%
Meropenem	1 mg/mL in water	0.170 mL	0.425 mg/L
Vancomycin	50 mg/mL in water	0.16 mL	20 mg/L
Amphotericin B	10 mg/mL in DMSO ^b^	0.2 mL	5 mg/L

^a^ A volume of 400 mL of medium was used for 20 plates. ^b^ DMSO, dimethyl sulfoxide.

## Data Availability

The datasets used and/or analyzed during the present study are available from the corresponding author upon reasonable request.

## Supplementary Materials

The following supporting information can be downloaded at https://www.mdpi.com/article/10.3390/antibiotics14050480/s1, Figure S1: Color change in the media with different fermenter and non-fermenter species. Left plate picture corresponds to the original color of the plate before bacterial growth. (A) *Stenotrophomonas maltophilia*, (B) *Acinetobacter baumannii*, (C) *Pseudomonas aeruginosa*, (D) *Klebsiella pneumoniae*, and (E) *Escherichia coli*. Table S1: Raw data of the evaluation step for meropenem. Table S2: Raw data of the clinical evaluation with 130 clinical specimens (TBA and BAL).
