# Supplementary material for: A Selective Chromogenic Medium for Detecting Meropenem-Resistant Pseudomonas aeruginosa in Respiratory Samples
Source: Antibiotics (Basel). 2025 May 9;14(5):480. doi: 10.3390/antibiotics14050480 (PMC12108385; doi:10.3390/antibiotics14050480)
Supplement: Supplementary file 1 [file antibiotics-14-00480-s001.zip › Figure S1.pdf]

Figure S1.

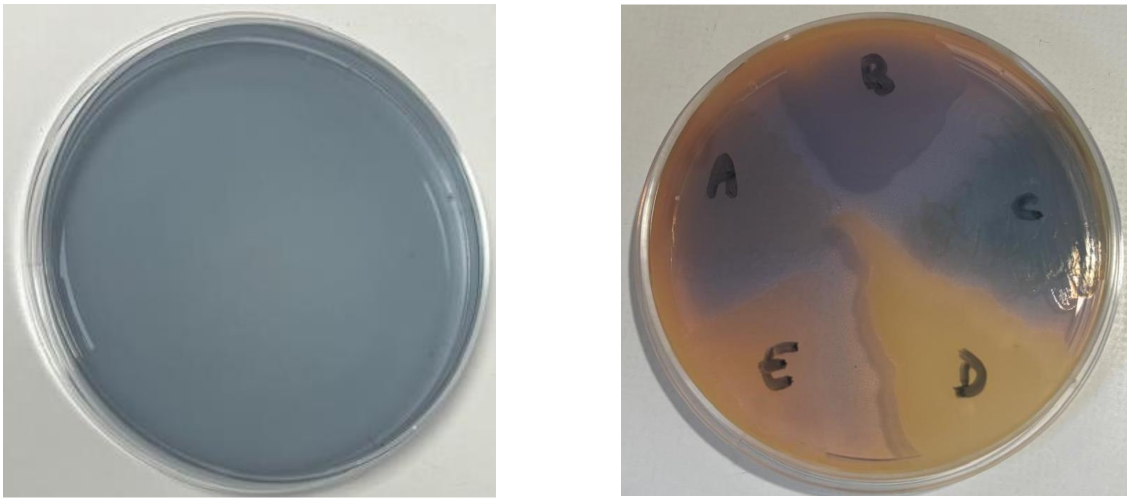

Supplementary Figure S1. Colour change of the media with different fermenter and non-fermenter species. Left plate picture correspond to the original colour of the plate before bacterial growth. (A) *Stenotrophomonas maltophilia*, (B) *Acinetobacter baumannii*, (C) *Pseudomonas aeruginosa*,(D) *Klebsiella pneumoniae*, and (E) *Escherichia coli.*
