# Supplementary material for: A Selective Chromogenic Medium for Detecting Meropenem-Resistant Pseudomonas aeruginosa in Respiratory Samples
Source: Antibiotics (Basel). 2025 May 9;14(5):480. doi: 10.3390/antibiotics14050480 (PMC12108385; doi:10.3390/antibiotics14050480)
Supplement: Supplementary file 1 [file antibiotics-14-00480-s001.zip › Table S1.pdf]

Table S1. Raw data of the evaluation step for meropenem.

STRAIN	10^4	10^5	10^6	10^7	10^8	MIC (mg/L)
**PAR1**	Y	Y	Y	Y	Y	16
**PAR100**	Y	Y	Y	Y	Y	16
**PAR101**	Y	Y	Y	Y	Y	32
**PAR102**	Y	Y	Y	Y	Y	4
**PAR103**	Y	Y	Y	Y	Y	**2**
**PAR104**	Y	Y	Y	Y	Y	16
**PAR105**	N	N	N	N	N	1
**PAR106**	Y	Y	Y	Y	Y	**2**
**PAR108**	Y	Y	Y	Y	Y	8
**PAR109**	Y	Y	Y	Y	Y	32
**PAR110**	Y	Y	Y	Y	Y	16
**PAR115**	Y	Y	Y	Y	Y	16
**PAR116**	Y	Y	Y	Y	Y	64
**PAR117**	Y	Y	Y	Y	Y	32
**PAR118**	Y	Y	Y	Y	Y	64
**PAR119**	Y	Y	Y	Y	Y	16
**PAR120**	Y	Y	Y	Y	Y	8 (32)
**PAR126**	N	N	N	N	Y	1
**PAR2**	Y	Y	Y	Y	Y	4
**PAR200**	Y	Y	Y	Y	Y	>64
**PAR201**	Y	Y	Y	Y	Y	16
**PAR202**	N	N	N	N	Y	0.5
**PAR203**	Y	Y	Y	Y	Y	32
**PAR205**	Y	Y	Y	Y	Y	64
**PAR208**	Y	Y	Y	Y	Y	16

**PAR209**	Y	Y	Y	Y	Y	16
**PAR210**	Y	Y	Y	Y	Y	16
**PAS244**	N	N	N	N	N	<0.125
**PAR301**	Y	Y	Y	Y	Y	8
**PAR302**	N	N	N	N	N	1
**PAR36**	N	N	N	N	N	0.5
**PAR39**	Y	Y	Y	Y	Y	8
**PAR40**	Y	Y	Y	Y	Y	16
**PAR44**	Y	Y	Y	Y	Y	8
**PAR47**	Y	Y	Y	Y	Y	8
**PAR49**	Y	Y	Y	Y	Y	16
**PAR5**	Y	Y	Y	Y	Y	16
**PAR50**	Y	Y	Y	Y	Y	16
**PAR51**	Y	Y	Y	Y	Y	32
**PAR52**	N	N	N	Y	Y	**4**
**PAR53**	Y	Y	Y	Y	Y	8
**PAR54**	Y	Y	Y	Y	Y	16
**PAR55**	N	N	N	N	N	1
**PAR56**	Y	Y	Y	Y	Y	4
**PAR57**	Y	Y	Y	Y	Y	16
**PAR59**	Y	Y	Y	Y	Y	8
**PAR6**	Y	Y	Y	Y	Y	8
**PAR60**	Y	Y	Y	Y	Y	16
**PAR61**	N	N	N	N	N	<0.125
**PAR82**	N	N	N	N	N	0.25
**PAR83**	N	N	N	N	N	2
**PAR84**	Y	Y	Y	Y	Y	4

**PAR87**	N	N	N	N	N	2
**PAR88**	N	N	N	N	N	2
**PAR89**	Y	Y	Y	Y	Y	**2**
**PAR9**	Y	Y	Y	Y	Y	16
**PAR90**	Y	Y	Y	Y	Y	16
**PAR91**	Y	Y	Y	Y	Y	16
**PAR97**	Y	Y	Y	Y	Y	16
**PAR98**	N	N	N	N	N	1
**PAR99**	N	N	N	N	N	0.5
**PARX1**	Y	Y	Y	Y	Y	16
**PAS 34**	N	N	N	N	Y	**2**
**PAS1**	N	N	N	N	N	0.5
**PAS10**	N	N	N	N	N	0.5
**PAS11**	N	N	N	N	N	<0.125
**PAS12**	N	N	N	N	N	<0.125
**PAS13**	N	N	N	N	N	<0.125
**PAS15**	N	N	N	N	N	<0.125
**PAS16**	N	N	N	N	N	<0.125
**PAS17**	Y	Y	Y	Y	Y	**2**
**PAS2**	N	N	Y	Y	Y	**0.5**
**PAS200**	N	N	N	N	N	1
**PAS201**	N	N	N	N	N	<0.125
**PAS203**	N	N	N	N	N	<0.125
**PAS207**	N	N	N	N	N	0.5
**PAS208**	N	N	N	N	Y	2
**PAS209**	N	N	N	N	N	2
**PAS210**	N	N	N	N	N	0.25

**PAS211**	N	N	N	N	N	0.25
**PAS212**	N	N	N	N	N	2
**PAS213**	N	N	N	N	N	1
**PAS214**	N	N	N	N	N	0.5
**PAS215**	N	N	N	N	N	1
**PAS216**	N	N	N	N	N	<0.125
**PAS217**	N	N	N	N	N	0.25
**PAS218**	N	N	N	N	N	0.25
**PAS219**	N	N	N	N	N	0.125
**PAS220**	N	N	N	N	N	0.25
**PAS221**	N	N	N	N	N	<0.125
**PAS227**	N	N	N	N	N	1
**PAS234**	N	N	N	N	N	1
**PAS236**	N	N	N	N	N	<0.125
**PAS239**	Y	Y	Y	Y	Y	**2**
**PAS24**	N	N	N	N	N	0.25
**PAS240**	N	N	N	N	N	0.5
**PAS241**	Y	Y	Y	Y	Y	**1**
**PAS242**	N	N	N	N	N	1
**PAS243**	N	N	N	N	N	2
**PAS245**	N	N	N	N	N	<0.125
**PAS246**	N	N	N	N	N	0.5
**PAS247**	N	N	N	N	N	2
**PAS248**	N	N	N	N	N	1
**PAS250**	N	N	N	N	N	0.5
**PAS251**	N	N	N	N	N	0.5
**PAS252**	N	N	N	N	N	125

**PAS253**	N	N	N	N	Y	**1**
**PAS254**	N	N	N	N	N	0.25
**PAS255**	N	N	N	N	Y	**0.5**
**PAS3**	N	N	N	N	N	0.5
**PAS30**	N	N	N	N	N	0.25
**PAS31**	N	N	N	N	N	2
**PAS32**	N	N	N	N	N	0.5
**PAS33**	N	N	N	N	N	1
**PAS4**	N	N	N	N	N	1
**PAS400**	N	N	N	N	N	1
**PAS401**	N	N	N	N	N	1
**PAS402**	N	N	N	N	N	1
**PAS403**	N	N	N	Y	Y	**2**
**PAS405**	N	N	N	Y	Y	**2**
**PAS5**	N	N	N	N	N	<0.125
**PAS59**	N	N	N	N	N	2
**PAS6**	N	N	N	N	N	<0.125
**PAS60**	N	N	N	N	N	<0.125
**PAS63**	N	N	N	N	N	0.25
**PAS64**	N	N	N	N	N	2
**PAS65**	N	N	N	N	N	2
**PAS66**	N	N	N	N	N	<0.125
**PAS77**	N	N	N	N	Y	**2**
**PAS79**	N	N	N	N	N	1

Y, growth; N, not growth
