# Supplementary material for: A Selective Chromogenic Medium for Detecting Meropenem-Resistant Pseudomonas aeruginosa in Respiratory Samples
Source: Antibiotics (Basel). 2025 May 9;14(5):480. doi: 10.3390/antibiotics14050480 (PMC12108385; doi:10.3390/antibiotics14050480)
Supplement: Supplementary file 1 [file antibiotics-14-00480-s001.zip › Table S2.pdf]

Table S2. Raw data of the clinical evaluation with 130 clinical specimens (TBA and BAL)

NUMBER	ST[A]	BACTERIAL CONCENTRATION (CFU/ml)	Seletive medium	REPORT (CONVENTIONAL TECHNIQUES)	CONFIRMATORY ANALISIS (Identification from selective medium)	MIC MER (BMD[C])
BC-1	TBA	-	-			
BC-2	TBA	-	-			
BC-3	TBA	-	-			
BC-4	TBA	>10^6	>10^4	*Stenotrophomonas maltophilia*	*Stenotrophomonas maltophilia*	
BC-5	TBA	>10^6	-	*Haemophilus influenzae*		
BC-6	TBA	-	-			
BC-7	TBA	>10^6	-	*Klebsiella pneumoniae*		
BC-8	TBA	-	-			
BC-9	TBA	-	-			
**BC-10**	**TBA**	**>10^6**	**>10^6**	***Pseudomonas aeruginosa***	***Pseudomonas aeruginosa***	**8**
**BC-11**	**BAL**	**>10^6**	**>10^6**	***Pseudomonas aeruginosa***	***Pseudomonas aeruginosa***	**32**
BC-12	TBA	-	>10^4		*Stenotrophomonas maltophilia*	
BC-13	TBA	-	>10^4		*Stenotrophomonas maltophilia*	
BC-14	BAL	-	-			
BC-15	BAL	-	-			

BC-16	BAL	-	-			
BC-17	TBA	10^4	-	*Staphylococcus aureus*		
BC-18	BAL	-	-			
BC-19	BAL	-	-			
BC-20	BAL	>10^6	>10^6	*Stenotrophomonas maltophilia*	*Stenotrophomonas maltophilia*	
BC-21	BAL	-	-			
BC-22	BAL	-	-			
BC-23	BAL	-	-			
BC-24	BAL	-	-			
BC-25	BAL	-	-			
BC-26	TBA	-	-			
BC-27	BAL	>10^4	-	*Escherichia coli, Staphylococcus aureus*		
BC-28	BAL	-	-			
BC-29	BAL	-	-			
BC-30	BAL	>10^6	>10^6	*Stenotrophomonas maltophilia*	*Stenotrophomonas maltophilia*	
BC-31	BAL	10^5	-	*Staphylococcus aureus*		
BC-32	BAL	-	-			
BC-33	BAL	-	-			
BC-34	TBA	>10^6	-	*Escherichia coli*		

BC-35	TBA	>10^6	-	*Klebsiella pneumoniae*		
BC-36	TBA	-	-			
BC-37	TBA	-	-			
BC-38	TBA	-	-			
BC-39	TBA	-	-			
BC-40	TBA	-	-			
BC-41	TBA	>10^6	>10^4	*Citrobacter koseri*	*Stenotrophomonas maltophilia*	
BC-42	TBA	-	-			
BC-43	TBA	-	-			
BC-44	TBA	-	-			
BC-45	TBA	-	-			
**BC-46**	**TBA**	**10^4**	**-**	***Pseudomonas aeruginosa***		**<=0.12**
BC-47	TBA	-	-			
BC-48	TBA	-	-			
BC-49	TBA	>10^6	>10^5	*Stenotrophomonas maltophilia*	*Stenotrophomonas maltophilia*	
BC-50	TBA	-	-			
BC-51	BAL	-	-			
BC-52	TBA	10^4	-	*Haemophilus influenzae*		
BC-53	BAL	-	-			
BC-54	BAL	>10^6	-	*Klebsiella pneumoniae*		

BC-55	BAL	-	-			
**BC-56**	**TBA**	**>10^6**	**>10^6**	***Pseudomonas aeruginosa***	***Pseudomonas aeruginosa***	**8**
BC-57	TBA	>10^6	-	*Proteus mirabilis*		
BC-58	TBA	-	-			
BC-59	TBA	-	-			
BC-60	TBA	-	-			
BC-61	TBA	>10^6	-	*Serratia marcescens*		
**BC-62**	**TBA**	**>10^6**	**-**	***Pseudomonas aeruginosa***		**2**
BC-63	TBA	-	-			
BC-64	TBA	-	10^5		*Stenotrophomonas maltophilia*	
BC-65	TBA	>10^6	-	*Haemophilus influenzae*		
BC-66	TBA	-	-			
BC-67	TBA	>10^6	-	*Staphylococcus aureus*		
BC-68	TBA	-	-			
BC-69	TBA	-	-			
BC-70	TBA	-	-			
BC-71	TBA	-	-			
BC-72	BAL	>10^6	-	*Klebsiella pneumoniae*		
BC-73	TBA	-	-			
BC-74	BAL	-	-			

BC-75	BAL	>10^6	-	*Staphylococcus aureus, Klebsiella pneumoniae*		
BC-76	BAL	10^4	10^5	*Stenotrophomonas maltophilia, Haemophilus influenzae*	*Stenotrophomonas maltophilia*	
BC-77	BAL	-	-			
BC-78	TBA	-	-			
BC-79	BAL	>10^6	-	*Enterobacter cloacae*		
BC-80	TBA	-	-			
BC-81	TBA	>10^6	>10^6	*Streptococcus pneumoniae*	*Burholderia gladioli*	
BC-82	TBA	-	-			
BC-83	BAL	-	-			
**BC-84**	**BAL**	**>10^4**	**10^5**	***Pseudomonas spp***	***Pseudomonas rhodesiae***	**8**
BC-85	BAL	-	-			
BC-86	BAL	>10^6	10^4	*Enterococcus faecalis*	*Stenotrophomonas maltophilia*	
BC-87	BAL	-	-			
BC-88	BAL	-	-			
BC-89	BAL	-	-			
BC-90	BAL	-	-			
BC-91	BAL	-	-			
BC-92	BAL	-	-			

BC-93	BAL	-	-			
**BC-94**	**BAL**	**-**	**>10^6**		***Pseudomonas fluorescens***	**32**
BC-95	BAL	-	-			
BC-96	BAL	-	-			
BC-97	BAL	>10^5	-	*Staphylococcus aureus*		
**BC-98**	**BAL**	**>10^4**	**-**	***Pseudomonas aeruginosa***		**<=0.12**
BC-99	BAL	-	-			
BC-100	BAL	-	-			
BC-101	BAL	-	-			
BC-102	BAL	>10^5	-	*Klebsiella aerogenes*		
BC-103	BAL	-	-			
BC-104	BAL	-	-			
BC-105	BAL	>10^5	-	*Escherichia coli*		
BC-106	BAL	-	-			
BC-107	BAL	-	-			
BC-108	BAL	-	-			
**BC-109**	**BAL**	**-**	**10^5-10^6**		***Pseudomonas fluorescens***	**2**
BC-110	BAL	>10^4	>10^6	*Stenotrophomonas maltophilia*	*Stenotrophomonas maltophilia*	
**BC-111**	**TBA**	**>10^5**	**>10^6**	***Pseudomonas aeruginosa***	***Pseudomonas aeruginosa***	**8**
BC-112	TBA	>10^5	-	*Klebsiella pneumoniae*		

BC-113	TBA	-	-			
BC-114	TBA	-	-			
BC-115	TBA	-	-			
BC-116	TBA	-	-			
**BC-117**	**TBA**	**>10^5**	**>10^6**	***Pseudomonas aeruginosa***	***Pseudomonas aeruginosa***	**8**
BC-118	TBA	>10^5	10^5	*Staphylococcus aureus*	*Stenotrophomonas maltophilia*	
BC-119	TBA	-	-			
BC-120	TBA	-	10^6		*Enterobacter cloacae*	
BC-121	TBA	-	-			
BC-122	TBA	-	-			
BC-123	TBA	>10^5	-	*Haemophilus influenzae*		
**BC-124**	**TBA**	**>10^5**	**>10^6**	***Pseudomonas aeruginosa***	***Pseudomonas aeruginosa***	**16**
BC-125	BAL	-	-			
BC-126	BAL	-	-			
BC-127	BAL	10^4	-	*Escherichia coli, Klebsiella aerogenes*		
BC-128	BAL	-	-			
BC-129	BAL	-	-			
**BC-130**	**TBA**	**>10^5**	-	***Pseudomonas aeruginosa***		**<=0.12**

TBA, tracheobronchial aspirate; BAL, bronchoalveolar lavage; MER, meropenem.
A: Specimen Type, B: Treatment of the patient when clinical sample was isolated (only for *P. aeruginosa*), C: Broth microdilution method.
